# Transplantation of Neural Progenitor Cells Derived from Stem Cells from Apical Papilla Through Small-Molecule Induction in a Rat Model of Sciatic Nerve Injury

**DOI:** 10.1007/s13770-024-00648-y

**Published:** 2024-06-21

**Authors:** Junhao Koh, Junqing Liu, Chi Him Poon, Jun Kang, Mohammed S. Basabrain, Lee Wei Lim, Chengfei Zhang

**Affiliations:** 1 Restorative Dental Sciences, Endodontology, Faculty of Dentistry, The University of Hong Kong, Hong Kong, China; 2 School of Biomedical Sciences, Li Ka Shing Faculty of Medicine, The University of Hong Kong, Pokfulam, Hong Kong, China; 3 Restorative Dental Sciences, Faculty of Dentistry, Umm Al-Qura, University, Makkah, Saudi Arabia

**Keywords:** SCAPs, Stem cells, Peripheral nerve regeneration, Cell sheet engineering, Nerve conduit

## Abstract

**Background:**

Stem cell-based transplantation therapy holds promise for peripheral nerve injury treatment, but adult availability is limited. A cell culture protocol utilizing a small-molecule cocktail effectively reprogrammed stem cells from apical papilla (SCAPs) into neural progenitor cells, subsequently differentiating into neuron-like cells. This study aims to evaluate neural-induced SCAPs, with and without small-molecule cocktail, for sciatic nerve repair potential.

**Methods:**

A scaffold-free cell sheet technique was used to construct a three-dimensional cell sheet. Subsequently, this cell sheet was carefully rolled into a tube and seamlessly inserted into a collagen conduit, which was then transplanted into a 5 mm sciatic nerve injury rat model. Functional sciatic nerve regeneration was evaluated via toe spread test, walking track analysis and gastrocnemius muscle weight. Additionally, degree of sciatic nerve regeneration was determined based on total amount of myelinated fibers.

**Results:**

Small-molecule cocktail induced SCAPs enhanced motor function recovery, evident in improved sciatic function index and gastrocnemius muscle retention. We also observed better host myelinated fiber retention than undifferentiated SCAPs or neural-induced SCAPs without small-molecule cocktail. However, clusters of neuron-like cell bodies (surrounded by sparse myelinated fibers) were found in all cell sheet-implanted groups in the implantation region. This suggests that while the implanted cells likely survived transplantation, integration was poor and would likely hinder long-term recovery by occupying the space needed for host nerve fibers to project through.

**Conclusion:**

Neural-induced SCAPs with small-molecule cocktail demonstrated promising benefits for nerve repair; further research is needed to improve its integration and optimize its potential for long-term recovery.

## Introduction

Peripheral nerve injuries often result in restricted activity or life-long disability. Despite advances in neural tissue regeneration, nerve autograft microsurgery is still the most common therapeutic approach for restoring the structure and function of the damaged nerve. However, the feasibility of nerve autograft is often hampered by limited donor tissue, dimension discrepancies between donor and recipient nerve, and potential donor site morbidity [1, 2]. Stem cell-based transplantation therapy holds much promise for the treatment of peripheral nerve injuries. Although neural stem cells are considered the optimal candidate, they are disadvantaged by their limited availability in adults, the required invasive harvesting procedures, and potential donor-site morbidity. So far, obtaining sufficient stem cells for transplantation has been challenging.

Dental-derived stem cells, such as dental pulp stem cells (DPSCs), stem cells from apical papilla (SCAPs) and stem cells from human exfoliated deciduous teeth, are typical postnatal mesenchymal stem cells (MSCs) that possess self-renewal, clonal, and multipotent differentiation capacity. When triggered by appropriate microenvironmental factors, these cells can differentiate into multiple primary cell phenotypes, including neuronal, adipose, endothelial, chondrogenic, and smooth muscle cells [3]. For instance, SCAPs are excellent seed cells for tissue engineering due to their good survivability and high proliferative capacity [4]. They have been reported to significantly improve nerve regeneration and exert neuroprotective effects after injection into the injured sciatic nerve [5]. This was attributed to their brain-derived neurotrophic factor (BDNF) release rather than glial differentiation. The lack of glial differentiation could be due to the short study period, as the* in vitro* conversion of stem cells (including DPSCs) to Schwann-like cells typically takes 18–21 days [6–8]. However, the study did not report whether SCAPs underwent neural differentiation after transplantation or whether they retained their original stem cell state. Recently, we utilized a small-molecule cocktail adapted from Hu et al. [9] to successfully enhance the differentiation of DPSCs, SCAPs, and gingival mesenchymal stem cells (GMSCs) into the neural lineage within 14 days [10]. We subsequently demonstrated that this small-molecule cocktail could reprogram SCAPs into neural progenitor cells (NPCs) within 3 days, leading to further differentiation into neuron-like cells [11]. However, further research is required to determine the fate of neural-induced SCAPs with or without small-molecule cocktail following transplantation and to assess whether this process can aid nerve regeneration.

Direct cell transplantation has several shortcomings, such as low cell survival rate, insufficient cell accumulation, spontaneous differentiation to other lineages, and abnormal cell organization following transplantation [12, 13]. It has been proposed that the use of nerve conduits as scaffolds loaded with gel-carrying cells and/or growth factors could bridge the gap between the damaged nerve connections to support cell survival, adhesion, proliferation, migration, and differentiation, as well as allow biochemical communication across the nerve gap [14]. Although various biomaterial scaffolds seeded with stem cells have shown some efficacy in facilitating the regeneration of peripheral nerves, these scaffold-based approaches have some inherent drawbacks. An alternative scaffold-free approach using cell sheet technology [15] could successfully overcome some major challenges of scaffold-based methods, such as failure to mimic the natural extracellular matrix, lack of intercellular cross-talk, selective degradation, and remodeling. Additionally, self-assembled cell sheets have a more even cell distribution and higher cell densities, and the seeded cells have enhanced stability [15].

This study aimed to combine the neural induction of SCAPs using a defined small-molecule cocktail with cell sheet technology and nerve conduit for regenerating peripheral nerve tissues. The regenerative potential of cell sheets of NPCs derived from SCAPs was assessed by* in vivo* transplantation in a rat sciatic nerve injury model, which has been utilized in previous studies [16, 17].

## Materials and Methods

### SCAPs Culture

Human stem cells from apical papilla (SCAPs) were gifted by Dr. Anibal Diogenes at the University of Texas, Health Science Center, Department of Endodontics [18]. These cells were grown in alpha modified Minimum Essential Medium (A-MEM) containing 10% fetal bovine serum and 1% penicillin–streptomycin. The cells were kept at 37 °C in an incubator with 5% CO^2^. The medium was replaced every 2–3 days, and cells were subcultured when they reached 80% confluence.

### Chemical Cocktail-Based SCAP Differentiation into Neural Lineages

At passage six, SCAPs were seeded onto 6-well culture plates (20,000 cells/cm^2^). Neural induction followed the protocol by Hu et al. [9] with some modifications, such as shortening the duration from 8 to 7 days. When SCAPs reached 60% confluence, the medium was changed to neural induction medium (NI; consisting of DMEM/F12: Neurobasal A [1:1] with 0.5% [v/v] N2, 1% [v/v] B27, 100 mM cAMP, 20 ng/mL basic fibroblast growth factor) supplemented with/without the chemical cocktail VCRFSGY (0.5 mM valproic acid, 3 µM CHIR99021, 1 µM Repsox, 10 µM forskolin, 10 µM SP600125, 5 µM GO6983, 5 µM Y-27632). The culture medium was refreshed on the third day.

### Immunocytofluorescent of SCAP

Cells were fixed with 4% (v/v) paraformaldehyde in phosphate-buffered saline (PBS; pH 7.4) at room temperature for 20 min. Cells were then washed three times with PBS, permeabilized with PBS containing 0.5% (w/v) Triton X-100 for 5 min at 4 °C, and blocked in PBS containing 5% (w/v) bovine serum albumin (Sigma-Aldrich; Burlington, MA, USA) at room temperature for 1 h. The fixed samples were incubated with primary antibodies against β-Tubulin III (Tuj1; 1:500; cat. No. ab18207; Abcam, Cambridge, UK). The samples were washed in PBS and incubated with secondary Alexa Fluor 488-conjugated goat anti-rabbit (cat. No. ab150077; Abcam) for 1 h at room temperature in the dark, followed by washing with PBS. The cell nuclei were stained with DAPI for 5 min at room temperature and the samples were imaged using confocal microscopy (LSM710; Carl Zeiss AG, Jena, TH, Germany) at X200 magnification.

### Cell Sheet Formation

The SCAPs were seeded at 20,000 cells/cm^2^ in a 35 mm UpCell dish (NunC, Rochester, NY, USA). After reaching confluence, the original culture medium was discarded and replaced with A-MEM, NI, and neural induction medium with small molecules (SM), respectively. After 3 days of induction, SM was replaced with only the NI for an additional 2 days.

On day 5, the first layer of the cell sheet was washed once in warm Phosphate-buffered saline (PBS), and 100 µL of the appropriate medium was added before placing a membrane onto the cell layer at room temperature to allow the cell sheet to attach to the membrane (30 min for A-MEM group, 90 min for NI and SM groups). After washing with PBS and adding 100 µL of the appropriate medium, the second cell sheet was placed on the membrane with the first cell sheet layer on top of the second cell sheet. The UpCell dish containing the two layers of cell sheets was placed back into the incubator (37 °C, 5% CO^2^) for 90 min. During this period, a third cell sheet was attached to a membrane in a similar manner to the first cell sheet at room temperature. After 90 min, 1 mL of the appropriate medium at 37 °C was added for 3 min to detach the two-layer cell sheet from the membrane. Next, 700 µL of the appropriate medium at 37 °C was added to the two-layer cell sheet. The third cell sheet was then added on top of the two-layer cell sheet and then placed back into the incubator for 90 min. After incubation, 2 mL of the appropriate medium was added to detach the three-layer cell sheet from the membrane.

After removing the membrane, 1.5 mL of medium was removed, leaving 0.5 mL of medium in the dish to allow the cell sheet to detach from the UpCell dish at room temperature (excessive medium will result in the cell sheet curling prematurely). The cell sheet was curled into a cylinder by using curved, smooth-surfaced micro forceps while tilting the dish at a 45-degree angle. The remaining medium was replaced with 500 µL of fresh, appropriate medium, and the cell sheet cylinder was placed back into the incubator for 1 day before surgery.

### Animal Model of Severe Sciatic Nerve Injury

Sixty Male Sprague–Dawley rats (weighing approximately 250 g at the time of surgery) were housed in individual ventilated filtered cages (maximum of two rats per cage) under standard animal laboratory conditions with a controlled temperature of 25 °C, 12 h light/dark cycle, and food and water ad libitum. A 5 mm section of the left sciatic nerve was removed using diamond-coated micro scissors. A 7 mm long nerve guide (NeuraGen; Integra lifesciences, Princeton, NJ, USA) with an internal diameter of 1.5 mm was used to bridge the resulting gap. The rats were randomized into five experimental groups, with 12 rats in each group. Due to severe autotomy, the remaining rats in each group were as follows: empty nerve conduit (Empty) = 6, Reverse direction autograft (Reverse) = 4, cell sheet cultured in A-MEM (A-MEM) = 6, cell sheet cultured in NI (NI) = 7, and cell sheet cultured in neural induction with small-molecule cocktail (SM) = 5.

The nerve guide was split open horizontally with sharp micro scissors under sterile conditions in a culture hood. The cell sheet (trimmed to 5 mm using a surgical blade) or autografts soaked in saline were inserted into the nerve guide. After inserting the cell sheet or autograft into the middle of the nerve guide, the horizontal cut of the nerve guide was closed by suturing the opening with 8–0 nylon suture 1.5 mm away from the two ends. Next, 1 mm of the proximal and distal stumps of the sciatic nerve were each sutured at the lumen wall with 8–0 nylon suture and pulled into the nerve conduit. In the Reverse direction autograft group, the 5 mm excised nerve was inserted into the nerve guide in the opposite orientation to simulate a misaligned nerve recovery model. The muscle layer was sutured with 3–0 stitches (multifilament absorbable suture). The skin was clamped with 9 mm stainless steel reflex clips. All groups were treated daily with pharmaceutical-grade cyclosporine-A (10 mg/kg body weight) injected subcutaneously starting 24 h before surgery and fed paracetamol syrup (65 mg/kg body weight) until the end of the study.

### Toe Spread Test

The toe spread test was used to evaluate motor recovery at week 12 post-surgery according to previous protocols with some modifications [19, 20]. Toe spreading was assessed by photographing the animal's paws inside a clear acrylic container. The camera was positioned beneath the transparent base of the container to capture images of the plantar surface of the paws.

A five-level scoring system was employed to analyze the toe spread functionality: a score of 0 denotes no observable toe spread; 1 denotes one toe spread out; 2 denotes two toes spread out; 3 denotes all four toes separated, but to a lesser degree than the unaffected side; and 4 denotes normal toe spread. A score of 0 is assigned if the assessment cannot be conducted, for example, due to severe toe flexion contracture. Additionally, the severity of toe contracture was evaluated using a three-level scoring system: a score of 0 denotes no contracture; 1 denotes mild contracture with visible toe spread; and 2 denotes severe contracture without any discernible toe spread.

### Walking Track Analysis

The rats were tested in a modified elevated plus maze (75 cm long, 15 cm wide), elevated above the ground (75 cm). Two arms were guarded by a wall (5 cm high), and the other two arms had a shallow wall (3 cm high). Access to the closed arm was blocked by inserting two detachable walls (5 cm high) to form a straight, long path between the two open arms. The start of one side of the opened arm was blocked and covered at the center of the maze to form a black room. A piece of white paper (90 cm long, 15 cm wide) was placed on the bottom of the track. Chinese calligraphy ink was applied onto the plantar surface of the rat's hind feet with a brush, and the animal was allowed to walk down the track, leaving their hind footprints on the paper. Various measurements were obtained from the footprints, including: (1) the print length (PL), which is the distance from the heel to the third toe; (2) the toe spread (TS), which is the distance between the first and fifth toes; and (3) the intermediary toe spread (ITS), which is the distance between the second and fourth toes. These measurements were collected from both the experimental (E) and normal (N) sides. Rats were made to walk down the path until 3 complete gait movements without the animal pausing midway through the gait movements were recorded and used to calculate average SFI. The SFI was calculated as described by Bain et al. [21] according to the equation:

$$\mathrm{SFI }= -38.3 \left(\frac{{\text{EPL}}-{\text{NPL}}}{{\text{NPL}}}\right)+109.5 \left(\frac{{\text{ETS}}-{\text{NTS}}}{{\text{NTS}}}\right)+13.3 \left(\frac{{\text{EITS}}-{\text{NITS}}}{{\text{NITS}}}\right)-8.8$$

The tracks were recorded at baseline before surgery and at weeks 2, 4, 6, 8, 10, and 12 after surgery.

### Gastrocnemius Muscle Weight

Upon sacrificing the animals at week 12, the wet muscle weight of the gastrocnemius muscle on both the experimental and normal sides was measured using an electronic weighing machine. The wet weight ratios of experimental side in relation to the normal side were calculated to evaluate the functional recovery of sciatic nerve.

### Immunofluorescent Analysis of the Nerve Conduit

Nerve conduits were harvested and fixed in 4% (v/v) paraformaldehyde in PBS (pH 7.4) overnight at 4 °C. The conduits were cryoprotected sequentially in 15% and 30% sucrose in PBS (pH 7.4) for 2 days and then snap-frozen in liquid nitrogen before storing at − 80 °C. Before cryosectioning into 10 μm sections, the nerve conduit was split in half. One half was sectioned longitudinally, while the other half was sectioned transversely. Transverse sections were separated into the mid-section of the nerve conduit (mid), the contact point between the cell sheet and rat sciatic nerve stump (joint), and the rat sciatic nerve itself (connecting).

Fixed tissues were rehydrated in PBS for 10 min on ice, permeabilized with PBS containing 0.3% (w/v) Triton X-100 for 10 min on ice, and then blocked in PBS containing 4% (w/v) bovine serum albumin (Sigma-Aldrich) and 0.3% (w/v) Triton X-100 for 1 h at room temperature. The fixed samples were incubated with primary antibodies against S100 calcium-binding protein B (S100B; 1:500; cat. no. ab52642; Abcam) and β-Tubulin III (Tuj1; 1:500; cat. no. NB100-1612; Novus, Centennial, CO, USA) overnight at 4 °C. The slides were washed in PBS and incubated with secondary Alexa Fluor 488-conjugated goat anti-chicken (cat. No. ab150173; Abcam) and Alexa Fluor 647-conjugated goat anti-rabbit (cat. No. #4414; Cell Signaling, Danvers, MA, USA) for 1 h at room temperature. Finally, nuclear staining was performed by adding DAPI mounting medium (cat. No. ab104139; Abcam). Images were captured at X100 magnification using a confocal microscope, LSM 900 Airyscan 2 (Carl Zeiss AG).

The total number of myelinated fibers on each transverse section of the mid, joint, and connecting sections was calculated by creating a trained segmentation model using ZEISS Arivis cloud’s (Carl Zeiss Microscopy GmbH, Jena, TH, Germany) deep learning AI segmentation, specifically to recognize nerve fibers encased within a myelin sheet. The trained model was then applied to the image analysis module of ZEN 3.8 (Carl Zeiss Microscopy GmbH) for image segmentation and analysis for accurate counting of myelinated fibers within each section.

### Statistical Analysis

Data are presented as mean ± standard deviation (SD). Significant differences were assessed using one-way analysis of variance (ANOVA). Due to differences in the sample sizes across the groups in our study caused by severe autotomy, the Gabriel test was employed to evaluate the significance of the differences between groups. The correlation coefficients between the gastrocnemius muscle ratio and the total number of myelinated fibers in the connecting region were calculated using Pearson's correlation analysis. All statistical analyses were carried out using SPSS version 29.0 (Chicago, IL, USA).

## Results

### Characterization of SCAP Following Small-Molecule Induction

In our previous study, we demonstrated that stem cells from apical papilla (SCAPs) exposed to small molecule cocktail (SM) for 3 days has higher expression of neural progenitor markers, such as paired-box gene 6 (pax6) and Sry-related HMG box 2 (sox2), compared to undifferentiated SCAP (A-MEM) or neural induction medium without small molecule cocktail (NI) [11]. SCAP in both NI and SM group would then further differentiate into mature neuron with increased expression of mature neuronal markers, such as neurofilament protein (NFM), neuron-specific nuclear protein (NeuN) and microtubule-associated protein 2 (MAP2) from day 5–7 [11]. Our previous data suggested that SCAP were induced into a neural progenitor cell (NPC)-like state at day 3.

**Fig. 1:**
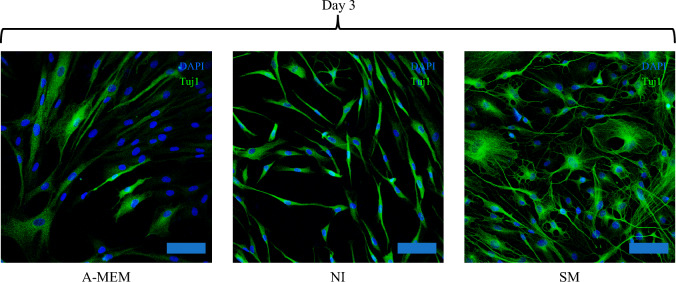
Expression of early neuronal marker of SCAP after 3 days of induction. Immunocytofluorescence for detection of tuj1 (green) expression in SCAP cultured in A-MEM, NI, and SM at day 3. Nuclei were counterstained with DAPI (blue). Scale bar = 100 µm. A-MEM, alpha modified Minimum Essential Medium; NI, Neural induction medium; SM, neural induction with small-molecule cocktail; Tuj1, β-Tubulin III

We further verify the neuronal fate of SCAP on day 3 of induction by examining the expression of neuronal marker, β-Tubulin III (Tuj1; Fig. 1), which is an earlier neuronal differentiation marker compared to MAP2 used in our previous study [11]. We noted mild expression of tuj1 in some of the undifferentiated SCAP, while all the cells in the NI and SM group have strong tuj1 expression at day 3.

### Cell Sheet Implantation and Autotomy Rate

**Fig. 2:**
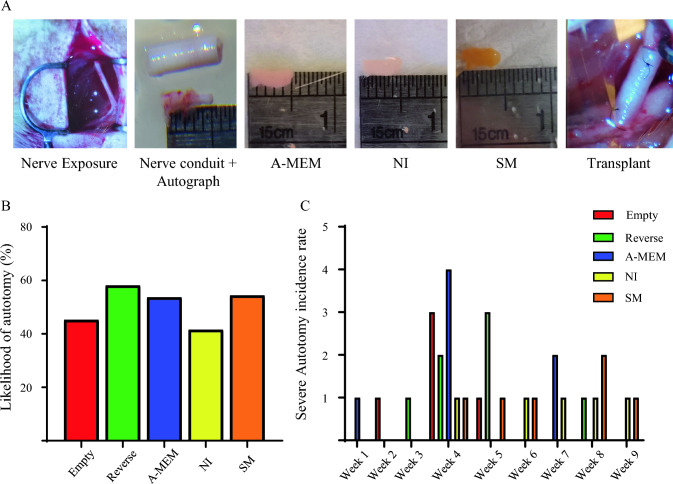
Delayed onset of autotomy in neural-induced SCAP-derived cell sheet groups. **A** Surgical procedure for inserting the cell sheet into a nerve conduit before transplanting into the 5-mm sciatic nerve gap. (From left to right: Exposure of left sciatic nerve; representative images of nerve conduit and excised nerve length; A-MEM cell sheet; NI cell sheet; SM cell sheet; the connected nerve in the nerve conduit.) Incidence of autotomy by group as shown by **B** percentage and **C** number of animals in each group by week. A-MEM, alpha modified Minimum Essential Medium; NI, Neural induction medium; SM, neural induction with small-molecule cocktail

The rat sciatic nerve injury model was established by excising 5 mm of the left sciatic nerve The length of cell sheets was about 5 mm before implantation. It was noted that the cell sheet cultured in the SM had a slightly yellowish color due to the SM cocktail. A 1 mm section of each end of the nerve stump was inserted into the 7-mm nerve conduit (Fig. 2A).

Transplantation of SCAPs-derived neural lineages delayed the onset of autotomy in the sciatic nerve injury rat model. As shown in Fig. 2B, the likelihood of autotomy was lower for the empty conduit group (45.5%) and NI group (41.6%), whereas the Reverse autograft group had the highest rate of autotomy (58%). The occurrence rate of autotomy by week revealed that the empty conduit and A-MEM groups peaked at week 4, followed by the Reverse autograft group at week 5, and SM group at week 8. The NI group had a more spread-out occurrence of autotomy, ranging from week 4–9, but with no distinct peak (Fig. 2C).

### Functional Evaluation of Cell Sheet Implantation

**Fig. 3:**
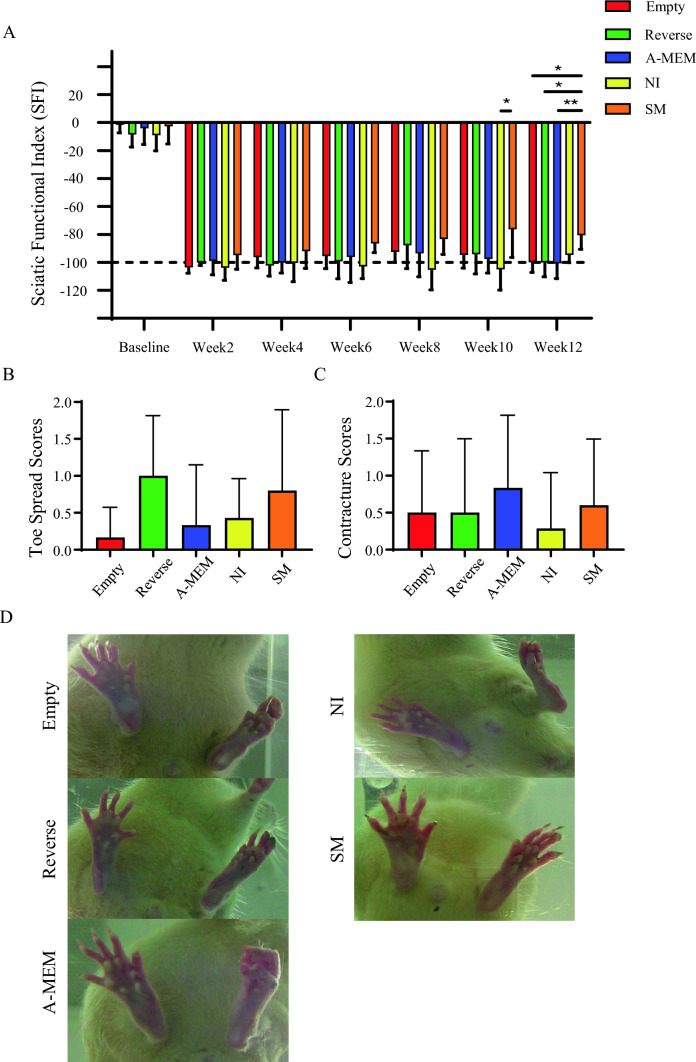
Assessment of functional recovery after cell sheet implantation. **A** At week 10, the SFI showed that the SM group had significantly more improvement than the NI group (*p < 0.05). Additionally, at week 12, the SM group outperformed the Empty (*p < 0.05), Reverse (*p < 0.05), and A-MEM (**p < 0.01) groups. **B** A bar chart of toe spread scores at week 12 post-injury revealed functional motor recovery in some animals in the Reverse and SM groups, but this was not statistically significant. Only the SM group demonstrated recovery in walking track analysis, as indicated by the SFI score. **C** Bar chart of the severity of chronic flexion contracture in each group at week 12 post-injury. **D** Representative images of toe spreading in each group showing the state of functional motor recovery of the foot at week 12. Data are presented as mean ± SD. A-MEM, alpha modified Minimum Essential Medium; NI, Neural induction medium; SFI, sciatic function index; SM, neural induction with small-molecule cocktail

SM-induced SCAPs-derived neural lineage transplantation significantly improved sciatic nerve function in sciatic nerve injury rats. Walking track analysis for assessing sciatic nerve recovery showed a notable improvement in the SM group at week 10 compared to the NI group (*p* < 0.05) and at week 12 compared to the Empty (*p* < 0.05), Reverse (*p* < 0.05), and A-MEM (*p* < 0.01) groups. Besides the NI group, all groups appeared to recover after surgery up to week 8, but their sciatic function index (SFI) scores worsened from week 8 onwards, except for the SM group, which continued to improve steadily up to week 12 (Fig. 3A).

No significant differences were observed in the toe spread or toe contracture scores; however, certain animals within the Reverse and SM groups demonstrated increased toe spreading compared to the other groups (Fig. 3B, D). Meanwhile, the A-MEM group was more prone to experiencing mild contracture (Fig. 3C, D).

**Fig. 4:**
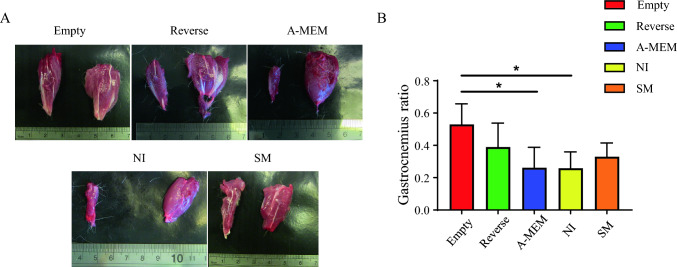
Gastrocnemius muscle retention following cell sheet implantation. **A** Representative left and right gastrocnemius muscle between groups and comparison of the wet gastrocnemius muscle ratio between left and right limb. **B** The bar chart shows that the Empty group retained significantly higher muscle mass than the A-MEM or NI groups (*p < 0.01). Data are presented as mean ± SD. A-MEM, alpha modified Minimum Essential Medium; NI, Neural induction medium; SM, neural induction with small-molecule cocktail

The degree of functional sciatic nerve recovery was measured by gastrocnemius muscle retention, as damage to the sciatic nerve can result in muscle atrophy due to inactivity following nerve severing. Our findings suggest that transplantation of neural-induced SCAPs with SM reduced gastrocnemius muscle atrophy in rats with sciatic nerve injury (Fig. 4A). The empty conduit group exhibited significantly greater recovery as determined by wet weight ratio of the gastrocnemius muscle compared to both the A-MEM and NI groups (*p* < 0.01). The SM group demonstrated recovery comparable to the Reverse autograft group and more significant than the A-MEM or NI groups, although this was not statistically significant (Fig. 4B).

### Sciatic Nerve Regeneration Following Cell Sheet Implantation

**Fig. 5:**
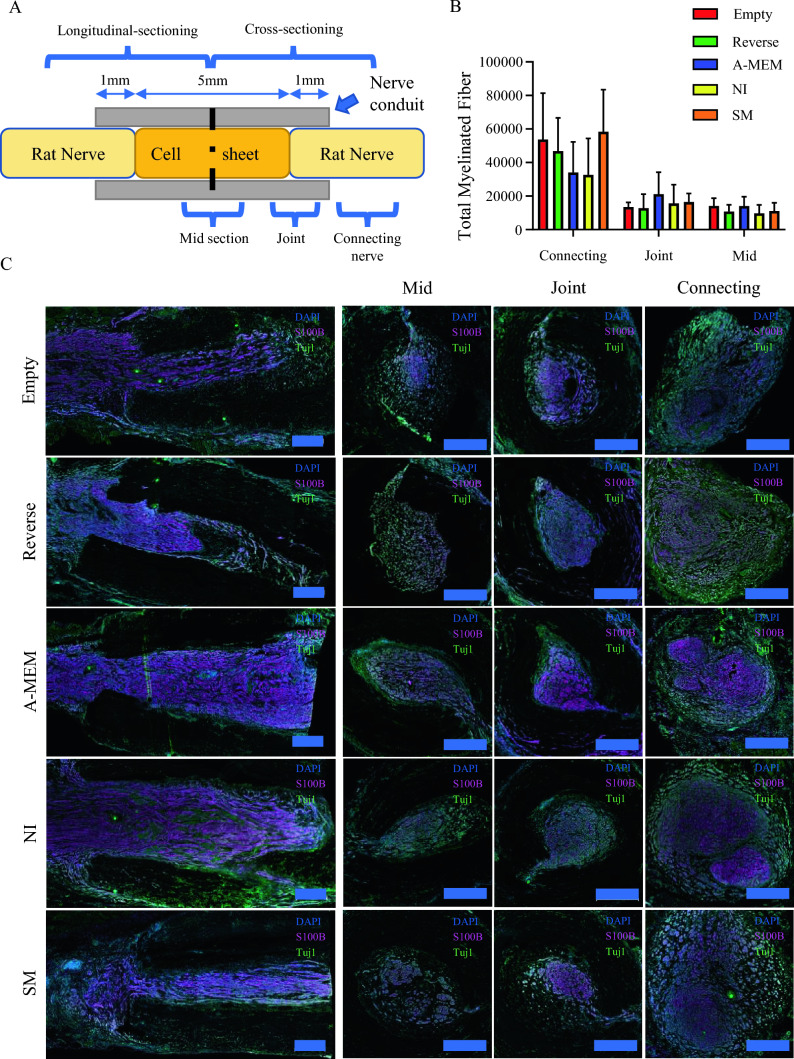
Effects of different cell sheet transplants on myelinated fiber regeneration. **A** Schematic diagram illustrating the sectional arrangement of the nerve for staining. **B** Bar chart showing total myelinated fibers. **C** Representative immunofluorescent staining of the operated sciatic nerve showing the longitudinal sectioning of the nerve and cross-sectioning of the mid-section of the conduits (Mid), the joint between the connecting nerve with the implanted section (Joint), and the connecting part of the nerve (Connecting) for each group. Nuclei were counterstained with DAPI (blue), Myelin-sheet were counterstained with S100b (purple) and neuronal fibers were counterstained with Tuj1(Green). Scale bar for all images = 500 µm. A-MEM, alpha modified Minimum Essential Medium; NI, Neural induction medium; S100b, S100 calcium-binding protein B; SM, neural induction with small-molecule cocktail; Tuj1, β-Tubulin III

Harvested nerves were cut in half and examined longitudinally and cross-sectionally to assess nerve regeneration (as illustrated in Fig. 5A). Although transplantation of the cell sheet did not significantly enhance the number of myelinated fibers in any section, the pattern of the bar chart of the total number of myelinated fibers in the connecting nerve section (Fig. 5B) closely resembled that of the gastrocnemius muscle ratio (Fig. 4B). Here, the number of myelinated fibers in the connecting nerve section of the SM group was almost comparable to the empty conduit group, closely followed by the Reverse autograft group. The A-MEM and NI groups had the lowest number of myelinated fibers in the connecting region. The correlation analysis revealed a significant relationship between the total number of myelinated fibers in the connecting region and the gastrocnemius muscle ratio (*r* = 0.386, *p* < 0.05). This finding suggests that preserving host myelinated fibers at the connecting region is associated with gastrocnemius muscle retention.

**Fig. 6:**
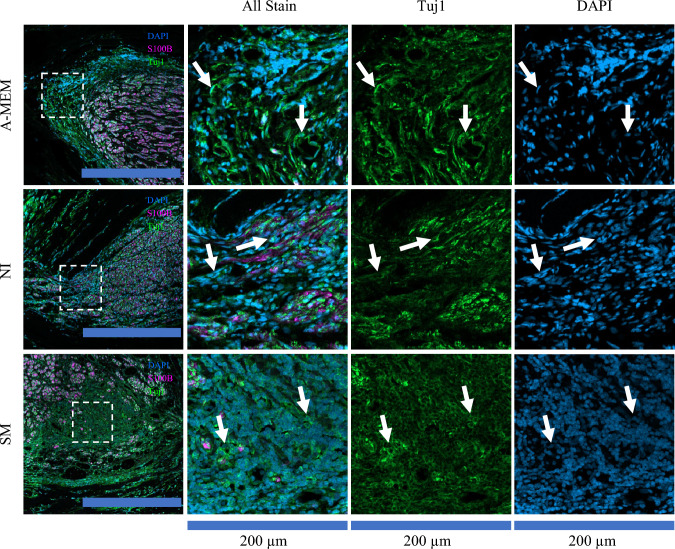
Cross-section of the mid-section showing transplanted cells surviving at 12 weeks. Clusters of non-myelinated cell bodies expressing Tuj1 were observed in the cell sheet groups (A-MEM, NI, and SM) but not in the Empty or Reverse groups. White arrows highlight the cell clusters. Nuclei were counterstained with DAPI (blue), Myelin-sheet were counterstained with S100b (purple) and neuronal fibers were counterstained with Tuj1(Green). The leftmost images are enlarged images from 200 µm × 200 µm dashed squares; the scale bar for the leftmost images = 500 µm. A-MEM, alpha modified Minimum Essential Medium; NI, Neural induction medium; S100b, S100 calcium-binding protein B; SM, neural induction with small-molecule cocktail; Tuj1, β-Tubulin III

Upon closer examination of the cell sheet-implanted samples, we observed the presence of non-myelinated Tuj1-positive cell body clusters predominantly in the mid-section, with some cases showing clusters in the joint section. Such cell clusters were not found in the Empty or Reverse groups. Under normal sciatic nerve regeneration, the cell bodies of mature motor neurons are located in the spine, and only the fibers of these neurons would extend to the limbs through the nerves to attempt to reconnect disconnected fibers (Fig. 6). Therefore, we suspect that these cell clusters represent surviving implanted SCAPs.

## Discussion

Nerve grafting is currently the gold standard and treatment for peripheral nerve injury. However, this approach is limited by donor site morbidity and the availability of autologous donor tissue [22, 23]. Various sources for stem cells have been considered to address the shortcomings of nerve grafting, which have shown promising results [24]. In particular, dental-derived stem cells have garnered much interest due to their easy accessibility [25]. A previous study comparing different dental-derived stem cells revealed that stem cells from apical papilla (SCAPs) promoted the most axon regeneration compared to dental pulp stem cells (DPSCs) and periodontal ligament stem cells (PDLSCs) [5]. However, such treatments have an inherent problem of the stem cells potentially differentiating into unwanted lineages that could hinder neuronal regeneration [26], highlighting the need to pre-differentiate stem cells towards a specific lineage before transplantation. In a prior study, we demonstrated that a chemical cocktail of small molecules VCRFSGY (0.5 mM valproic acid, 3 µM CHIR99021, 1 µM Repsox, 10 µM forskolin, 10 µM SP600125, 5 µM GO6983, 5 µM Y-27632), rapidly induced the differentiation of SCAPs into neural lineage cells. The small-molecule cocktail induced the transformation of SCAPs into neural progenitor cell (NPC)-like state by day 3, followed by further differentiation into neuron-like cells [11]. The robust expression of the early neuronal marker, β-Tubulin III (tuj1), in SCAPs cultured under neural induction medium (NI) or with small molecule cocktail (SM) conditions by day 3, as demonstrated in this study, confirms the successful initiation of neuronal differentiation in these two experimental groups before transplantation. Using a sciatic nerve injury rat model, the current study evaluated the regenerative potential of neural-induced SCAPs with and without SM.

The functional evaluation of the motor recovery indicated that animals implanted with cell sheets of SM-induced SCAPs progressively performed better in the walking track assessments, whereas the other groups showed little to no recovery. Furthermore, although SCAPs and NI SCAPs displayed significantly lower gastrocnemius muscle retention than the empty conduit group, the SM SCAPs group experienced less muscle atrophy compared to SCAPs or NI group, although the difference was not significant. The lack of functional recovery in undifferentiated SCAPs was surprising, given that it was previously demonstrated to improve recovery in both spinal cord injury [27] and sciatic chronic constriction injury [28] and was shown to promote the most axon regeneration when compared to rat Schwann cell, DPSCs, and PDLSCs [5]. The wet weight ratio of the SM SCAPs group was almost comparable to the Reverse autograft group, which simulates incorrect nerve alignment. A previous study demonstrated better muscle retention in mice transplanted with motor neurons derived from human induced pluripotent stem cells than those without transplants, although they did not examine walking performance [29]. Our results suggest that pre-differentiating SCAPs towards the neural lineage can provide functional benefits for sciatic nerve regeneration.

Intriguingly, although the empty conduit and Reverse autograft groups exhibited less gastrocnemius muscle atrophy and showed a certain degree of improvement in sciatic function index (SFI) until week 8, their walking test assessment deteriorated from weeks 10 to 12. The empty conduit group showed no sign of toe spread at week 12, whereas some animals in the Reverse autograft group exhibited signs of toe spread recovery. This implies that nerves likely reinnervated back to the gastrocnemius muscle in the empty conduit group to prevent atrophy, as the successful reinnervation of muscle is associated with lower muscle loss [30]. However, in our study, this recovery was insufficient to be functional, although we cannot definitively explain the deterioration in performance. Our study has several limitations, notably the absence of nerve fiber quantification, analysis of neuromuscular junction structures, assessment of muscle force, and acquisition of electrophysiological data. Such factors would contribute to a more comprehensive understanding of functional recovery and muscle atrophy.

Examining the total number of myelinated fibers, we observed a positive association between the number of myelinated fibers at the nerve connecting section and gastrocnemius muscle retention. However, we did not find significant differences between the groups for the number of myelinated fibers at each examined section (mid, joint, and connecting). Nevertheless, the empty conduit and SM groups had a comparable number of myelinated fibers at the nerve connecting section, which was higher than in the other groups. Additionally, large clusters of cell bodies exhibiting neuronal marker Tuj1 were identified in the cell sheet-implanted groups (including the undifferentiated SCAPs group), with the majority found around the mid-section, with some samples showing the marker in the joint section. These clusters were absent in the empty conduit and Reverse autograft groups. This finding suggests that, even without neural induction, undifferentiated SCAPs may differentiate toward a neural lineage after implantation for 12 weeks in the sciatic nerve injury model. Notably, very few myelinated fibers were seen running through these clusters. Unfortunately, we were unable to successfully label the human nucleus marker, which would have allowed us to determine with certainty the fate of implanted cells and whether they differentiated into other cell lineages, such as Schwann cells, or maintained their neuron-like characteristics. Nevertheless, the scarcity of myelinated fibers running through these clusters suggests these neuron-like clusters poorly integrate with the host nerve, which may present challenges for achieving full functional recovery. Despite the promising improvements in walking track, gastrocnemius muscle retention, and the number of myelinated fibers at the connecting section of the nerve observed in the SM group, such clusters would occupy the space and ultimately prevent the reinnervation of the severed region.

Autotomy rate, although not always discussed in sciatic nerve research, poses a significant challenge in such studies, particularly those using walking track analysis, as severe autotomy could render it impossible to quantify the SFI score [31–33]. A comparison of autotomy rates among different rat strains with severed versus repaired nerves revealed that Sprague–Dawley rats had a 100% autotomy rate with a mean onset of 3.5 weeks after nerve severing. However, if the nerve was immediately sutured back together, the rate dropped to 71% with a mean onset of 1.9 weeks [32]. In our study, a 5 mm nerve segment was excised and reconnected using a conduit with/without a cell sheet or a Reverse autograft. Autotomy rates were relatively similar across groups, ranging from 41% in the NI group to 58% in the Reverse autograft group. Compared to the previous study that reported an earlier onset of autotomy in repaired nerves, our study showed the onset varied among groups: empty conduit and SCAPs cell sheet groups exhibited earlier onset (peak at week 4), followed by the Reverse autograft group (peak at week 5), NI group (no peak), and the SM group (peak at week 8). Overall, the rate of autotomy was decreased in the Sprague–Dawley rats regardless of our treatment groups. The reduced autonomy in our study may be partially attributed to the collagen nerve conduits used to reconnect the nerves, which have been demonstrated to decrease autotomy severity and delay its onset [34]. However, it should be noted that the SM group exhibited the greatest autotomy onset delay and showed steady improvement in walking test assessments. Additionally, this group retained a degree of gastrocnemius muscle preservation, almost comparable to the Reverse autograft group.

Scaffold-free cell sheet formation has been employed in tendon repair [35] and for generating decellularized Schwann cell sheets for nerve regeneration (although this has not yet been tested in animal studies) [36]. To our knowledge, this approach has not been applied in sciatic nerve repair. The advantages of scaffold-free cell sheet formation include more uniform cell distribution, higher cell densities, and enhanced stability of the seeded cells [15]. Additionally, this method has been demonstrated to enable the stacking of different complementary cell sheets together to form a 3D construct [37]. Thus far, autografts have remained the gold standard for nerve injury repair, particularly when the repair area exceeds 5 mm. A clinical study showed that the success rate of nerve conduit alone was more varied and less reliable in such cases [38]. The scaffold-free method could allow researchers to fabricate more complex structures to mimic actual nerves, such as using electrical stimulation to dictate the direction of motor neurons co-cultured with Schwann cells [39]. Our results indicate that early induction of SCAPs towards neural lineage using a small-molecule cocktail is beneficial in promoting sciatic nerve recovery but likely not sufficient for full recovery in the long term.

Given that dental stem cells such as DPSCs have been shown to have the ability to differentiate into Schwann cells, it is plausible that SCAPs may also have this ability, although this has not yet been demonstrated [7]. Our next step will be to attempt to co-culture mature neurons and Schwann cells derived from SCAPs and then examine their peripheral nerve regeneration potential. Additionally, considering the high autotomy rate in Sprague–Dawley rats, it may be beneficial to use Lewis rats in future studies, as they have been reported to not self-mutilate following sciatic nerve injury [32].

## Data Availability

The datasets generated during and/or analysed during the current study are available from the corresponding author on reasonable request.
